# Arabinogalactan Proteins: Focus on the Role in Cellulose Synthesis and Deposition during Plant Cell Wall Biogenesis

**DOI:** 10.3390/ijms23126578

**Published:** 2022-06-13

**Authors:** Sue Lin, Yingjing Miao, Huiting Huang, Yuting Zhang, Li Huang, Jiashu Cao

**Affiliations:** 1 Institute of Life Sciences, College of Life and Environmental Science, Wenzhou University, Wenzhou 325035, China; 20451334008@stu.wzu.edu.cn (H.H.); 20461337006@stu.wzu.edu.cn (Y.Z.); 2 Biomedicine Collaborative Innovation Center of Zhejiang Province, Wenzhou University, Wenzhou 325035, China; 3 Laboratory of Cell & Molecular Biology, Institute of Vegetable Science, Zhejiang University, Hangzhou 310058, China; yjmiao@zju.edu.cn (Y.M.); lihuang@zju.edu.cn (L.H.); jshcao@zju.edu.cn (J.C.)

**Keywords:** arabinogalactan proteins, cell wall, cellulose synthesis, cellulose deposition, characteristics, classification, identification, biological function

## Abstract

Arabinogalactan proteins (AGPs) belong to a family of glycoproteins that are widely present in plants. AGPs are mostly composed of a protein backbone decorated with complex carbohydrate side chains and are usually anchored to the plasma membrane or secreted extracellularly. A trickle of compelling biochemical and genetic evidence has demonstrated that AGPs make exciting candidates for a multitude of vital activities related to plant growth and development. However, because of the diversity of AGPs, functional redundancy of AGP family members, and blunt-force research tools, the precise functions of AGPs and their mechanisms of action remain elusive. In this review, we put together the current knowledge about the characteristics, classification, and identification of AGPs and make a summary of the biological functions of AGPs in multiple phases of plant reproduction and developmental processes. In addition, we especially discuss deeply the potential mechanisms for AGP action in different biological processes via their impacts on cellulose synthesis and deposition based on previous studies. Particularly, five hypothetical models that may explain the AGP involvement in cellulose synthesis and deposition during plant cell wall biogenesis are proposed. AGPs open a new avenue for understanding cellulose synthesis and deposition in plants.

## 1. Introduction

Arabinogalactan proteins (AGPs) are a class of proteoglycan compounds that are widely present throughout the plant kingdom, as *Arabidopsis thaliana* (L.) Heynh., *Nicotiana tabacum* L., *Brassica napus* L., and maize (*Zea mays* L.) [1,2]. They are ubiquitous in all plant tissues and cells and found in cell walls, plasma membranes, and extracellular secretions of plants [3]. AGPs have been identified in a variety of angiosperms, gymnosperms, and lower plants (e.g., bryophytes and algae), such as rice (*Oryza sativa* L.), Chinese cabbage (*B. rapa* L.), *Picea abies* (L.) Karst., *Physcomitrella patens* (Hedw.) Bruch & Schimp, *Polytrichastrum formosum* (Hedw.) G.L.S.M, and *Ectocarpus siliculosus* (Dillw.) Lyngb. [4,5,6,7,8,9,10,11,12,13,14,15,16]. Several excellent reviews have summarized that AGPs are associated with vegetative growth, reproductive development, tissue regeneration, stress response, and other vital activities in plants [2,17,18,19,20,21,22,23,24,25,26,27,28,29]. However, the exact molecular mechanisms of AGP action in complicated biological processes are still unresolved and puzzling [28]. Here we describe, in detail, the characteristics, classification, identification, and biological functions of AGPs. Some progresses in understanding the synthesis and deposition of cellulose are briefly summarized. A focus is especially placed on the way AGPs might participate in cellulose synthesis and deposition during cell wall biogenesis, and as a result, five hypothetical models of AGP action are proposed.

## 2. Characteristics, Classification, and Identification of AGPs

### 2.1. Characteristics

AGPs belong to a superfamily of hydroxyproline (Hyp)-rich glycoproteins (HRGPs), which also includes extensins (EXTs), proline (Pro)-rich proteins (PRPs), and solanaceous lectins [30]. AGPs consist of a Hyp-rich core protein backbone with a molecular mass of about 60–300 kDa, decorated by arabinose (Ara)- and galactose (Gal)-rich polysaccharide units *O*-glycosidically linked to Hyp residues [1,2,19,30]. Their carbohydrate moieties typically account for more than 90% of their molecular mass [1,30].

All protein backbone precursors of AGPs are expected to have an N-terminal signal peptide sequence and a domain of variable length rich in Pro, alanine (Ala), serine (Ser), and threonine (Thr) (PAST) [31,32,33,34]. In addition, a prerequisite for defining an AGP is to possess AG glycomodules (amino acids regularly arranged as Ala-Pro, Pro-Ala, Ser-Pro, and Thr-Pro repeats, without EXT glycomodules (e.g., Ser-Pro_2-4_)) [35]. The presence of a C-terminal glycosylphosphatidylinositol (GPI) anchor signal sequence in most AGPs provides additional support for the identification of an AGP [35]. The maturation of AGP molecules involves proper post-transcriptional modifications, which mainly leads to the removal of N-terminal signal peptide, optional attachment of C-terminal GPI anchor, hydroxylation of Pro residues into Hyp residues, and arabinogalactan (AG) *O*-glycosylation [18,36,37].

Given that AGPs are typically at least 90% carbohydrate moieties by mass, carbohydrate moieties probably determine the interactive molecular surface, postsecretory fate, and ultimately, the functions of AGPs [3,38,39,40]. These carbohydrate units vary in size from 30–150 carbohydrate residues, but exhibit a type II AG polysaccharide structure, which is *O*-glycosidically linked to protein backbones at Hyp residues [2,19]. The type II AG polysaccharide structure consists of a *β*-1,3-galactose backbone decorated with *β*-1,6-galactose side chains, which are further modified by *α*-arabinose side chains and other relatively less abundant carbohydrates, such as *β*-(methyl)glucuronic acid (GlcA), *α*-rhamnose (Rha), and *α*-fucose [41]. Especially, 3-*O*-methyl-rhamnose as a terminal monosaccharide and galactan core highly branched with the unusual branching point 1,2,3-linked galactose, never found in AGPs of angiosperms, have been uniquely found in moss [9]. The AG polysaccharide consensus structure has the theoretical molar ratios: Gal_5_, Ara_6_, GlcA_2_, and Rha_2_ [42,43]. The polydispersity of AGPs is mainly caused by the variable number of repetitive AG subunits (repetitive glycomotifs of ~15 sugar residues) rather than the heterogeneity [43].

In the last decade, the identification of some AGPs lacking signal peptides in Arabidopsis, wheat (*Triticum aestivum* L.), and rice and some AGPs potentially lacking well-identified *O*-glycosylation sites in poplar (*Populus trichocarpa* Torrey & A. Gray ex Hooker), Chinese cabbage, wheat, and rice through bioinformatics approaches is challenging our conventional concept to define an AGP [7,35,44,45,46], without excluding that there are several wild species, including crop wild relatives, of which we have no data, not only for AGPs, but even for their chemical composition [47,48,49].

### 2.2. Classification

Based on amino acid compositions, size, and specific amino acid motifs of protein backbones, AGPs can be divided into classical AGPs, AG-peptides, chimeric AGPs (CAGPs), and nonclassical AGPs, as classified by Showalter et al. [35]. Classical AGPs are usually composed of an N-terminal signal peptide sequence, a central region with biased amino acid compositions of at least 50% PAST and putative AG glycomodules, and a C-terminal GPI-anchored signal [31,32,33,34]. Some classical AGPs containing a lysine (Lys)-rich insert within the PAST-rich domain are defined as Lys-rich AGPs [50]. Those AGPs that are between 50 and 90 amino acids in length with biased amino acid compositions of at least 35% PAST and have a predicted signal peptide sequence at their N-terminus are called AG peptides [11,35]. CAGPs are longer than 90 amino acids in length and possess other sequence motifs as well as putative AG glycomodules. CAGPs could be further classified into several subfamilies based on other specific protein domains, such as fasciclin domain in fasciclin-like AGPs (FLAs), nonspecific lipid-transfer protein (nsLTP) domain in xylogen-like AGPs (XYLPs), plastocyanin-like (PCNL) domain in plastocyanin-like AGPs (PLAs/PAGs), protein kinase domain in PK-like CAGPs, and formin homology 2 domain in FH2-like CAGPs [11,33,34,35,51,52,53,54]. In addition, some CAGPs harbor both characteristic domains of AGPs and EXTs, which have been identified and defined as AGP/EXT hybrids (HAE) [35].

### 2.3. Identification

The use of the beta-glucosyl Yariv reagent (*β*-Yariv; binds and perturbs AGPs) or a set of AGP-specific monoclonal antibodies (mABs; recognize AGP carbohydrate epitopes) is a traditional way to identify AGPs in plant tissues; however, such generalities are too narrow to account for all AGPs [2,36,55,56]. To date, genomes of many plant species have been sequenced, which has enabled the identification of AGPs using bioinformatics approaches. Based on the arrangement of amino acid composition, a series of methods have successively developed to search for AGP-coding genes, such as “amino acid bias” program, hidden Markov models, BIO OHIO, and python script “Finding-AGP” [11,31,35,57]. In the meanwhile, the basic local alignment search tool also helps to search for CAGPs that are not processed by other programs [34,58]. To date, a total of 151 and 282 putative AGPs have been identified in Arabidopsis and rice, respectively [11,12,31,34,35,53,57,58,59].

## 3. Biological Functions of AGPs

Current studies took advantage of immunocytochemistry, reverse genetics, transcriptomics, proteomics, and molecular approaches to explore biological functions of AGPs in a broad range of plants [24,60,61]. Indeed, such experimental approaches have demonstrated that AGPs are implicated in various biological processes, including cell expansion and differentiation, embryogenesis, seed germination, root development, sexual reproduction, fruit ripening, biotic and abiotic stress response, signal transduction, and response to multiple plant hormones [2,17,18,19,20,21,22,23,24,25,26,27,28,29,62].

In the following, we summarize the expression patterns, genetic analyses, and biological functions of AGPs that have been characterized so far (Table 1). It is shown that AGPs are expressed in almost all plant tissues and organs and widely participate in plant growth and reproduction. In addition, we highlight advances in understanding AGPs involved in the synthesis and deposition of cellulose components during cell wall biogenesis.

## 4. Involvement of AGPs in Cellulose Synthesis and Deposition during Plant Cell Wall Biogenesis

### 4.1. Cellulose Synthesis and Deposition during Plant Cell Wall Biogenesis

Plant cell walls are largely composed of cellulose, hemicelluloses, and pectins, along with a small amount of proteins and other compounds [147,148,149]. As the most abundant and main load-bearing biopolymer of the cell wall, cellulose is synthesized by cellulose synthase (CesA) proteins, integral plasma membrane proteins arranged into a unique hexagonal rosette complex called the cellulose synthase complex (CSC) [149,150].

There are several articles that cover many aspects of cellulose biosynthesis, which include CSC assembly in the Golgi apparatus, trafficking of CSCs to the plasma membrane, relationship between cellulose deposition and the underlying cortical microtubules, and post-translational modification of CesAs [151,152,153,154,155,156,157,158,159,160,161,162,163]. Several excellent reviews have summarized the genes and enzymes related to the synthesis and deposition of cellulose [149,150,158,161,164,165,166]. What is drawing our attention is that some AGPs, especially FLAs, have also been involved in cellulose synthesis and deposition [164,167,168].

### 4.2. AGPs Implicated in Cellulose Synthesis and Deposition

It has been proposed that some AGPs contribute to different biological processes, such as fiber development, microspore formation, and root growth via their impacts on cellulose synthesis and deposition. Cotton fibers are highly specialized and extremely elongated single-cell trichomes from seed epidermis, which are mainly composed of cellulose (>90%) [169,170]. Abundant AGP carbohydrate epitopes have been detected during the formation of cotton fibers, and several fiber-preferential genes encoding FLAs were isolated from cotton (*Gossypium hirsutum* L.) [123,124,125,171], implying that AGPs are probably implicated in the synthesis of cellulose. Direct evidence that cross-linking of AGPs with *β*-Yariv inhibits cellulose deposition on cultured tobacco protoplasts also gives a hint that AGPs are related to cellulose deposition [172]. In an increasing volume of evidence, this assumption has been further supported by phenotyping of loss-of-function and gain-of-function mutants. RNA interference (RNAi) of *GhAGP4* inhibits fiber initiation and elongation in cotton and affects cellulose deposition of fiber cells. Suppression of *GhAGP4* downregulates the expression level of the cellulose biosynthesis-related gene *celA1*, providing the direct proof that FLAs may affect the cell wall synthesis through cellulose deposition [126]. Overexpression of *GhFLA1* in cotton promotes fiber elongation, whereas suppression of *GhFLA1* slows down fiber initiation and elongation. In addition, expression levels of the genes involved in cellulose biosynthesis are remarkably enhanced in the *GhFLA1* overexpression transgenic fibers, leading to a higher rate of cellulose. In contrast, the transcripts of these genes are dramatically reduced in *GhFLA1* RNAi transgenic fibers with a lower rate of cellulose [124]. The intine of nearly half of the pollen grains in *AtFLA3* RNAi transgenic plants appears to have some abnormalities, with an abnormal cellulose distribution, indicating that *AtFLA3* may affect the pollen wall development by influencing cellulose deposition [84]. *BcMF18* in *B. campestris*, encoding a classical AGP, is specifically expressed in pollen grains. Antisense transgenic pollen also shows intine layer development defects similar to *FLA3* RNAi transgenic plants [72]. The case in Arabidopsis with a T-DNA insertion mutation of *FLA1*, showing a change of cellulose deposition in *fla1*, is also in support of this view [108]. *AtFLA11*/*IRX13* and *AtFLA12* participate in the formation of secondary cell walls, and double mutant shows reduced cellulose content, increased cellulose microfibril angle (refers to the microfibril deviation in the cell wall layer from the long axis of the cell), and impaired structure and composition of cell walls [98,99,158,173]. *AtSOS5*/*AtFLA4* is found to cooperate in the cell wall sensing system and facilitate cellulose synthesis [38,109,110,111,112,113,114,115,116]. An *atfla16* mutant shows that loss of FLA16 leads to reduced levels of cellulose and reduced stem length [100]. Unfortunately, because AGPs form a large family and a single-knockout mutant rarely results in a detectable phenotype, the precise functions of AGPs and their mechanisms of action in cellulose biosynthesis remain unclear.

In this current work, we propose some assumptions about the potential mechanisms of AGPs to participate in complex biological processes via their impacts on cellulose synthesis and deposition based on previous studies.

#### 4.2.1. AGPs Are Involved in Cellulose Synthesis via the 1-Aminocyclopropane-1-Carboxylic Acid (ACC)-Mediated Pathway

ACC is the direct precursor of ethylene, and the majority of the regulatory mechanisms of ethylene biosynthesis act at the level of ACC production by ACC synthases (ACSs) [174]. In addition to its role as the central molecule of ethylene biosynthesis, ACC is also capable of functioning in some biological processes via an ethylene-independent way. Tsang et al. found that the effect of ACC on primary root elongation in acute response to cell wall stress was partially independent of its conversion to ethylene or ethylene signaling in Arabidopsis [175]. The inhibition of cell elongation caused by disturbed cellulose biosynthesis can be fully restored in the short term by blocking ACC signaling despite the presence of visible cell wall damage [175].

**Figure 1:**
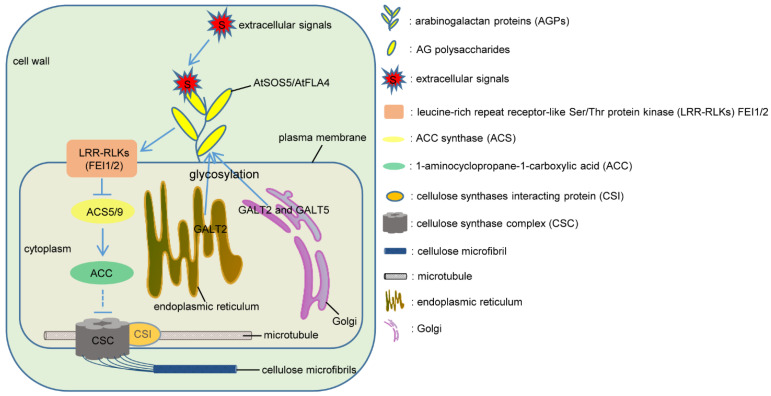
A hypothetical model of AGP involvement in cellulose synthesis via the 1-aminocyclopropane-1-carboxylic acid (ACC)-mediated pathway. AGPs may sense extracellular signals by carbohydrate moieties and transmit signals to some receptor kinases, thereby regulating cell wall formation by promoting cellulose synthesis through an ethylene-independent ACC pathway. Cellulose microfibrils are synthesized by cellulose synthase complexes (CSCs) that are present at the plasma membrane. GALT2 localized to the endoplasmic reticulum (ER) and the Golgi and GALT5 localized to Golgi vesicles function in AGP *O*-glycosylation [40]. AtSOS5/AtFLA4, FEI1, and FEI2 are localized to the plasma membrane [109,110]. The GALT2 GALT5/AtSOS5/FEI1 FEI2 pathway is represented according to Basu et al. [116].

It has been suggested that ACC might also be involved in AGP-related cell wall formation via an ethylene-independent pathway. A loss-of-function mutant of *AtSOS5*/*AtFLA4*, which lacks a GPI-anchored extracellular FLA, presents an impaired root growth and radial root tip swelling phenotype under high salt conditions [38,109,114,116]. What is particularly interesting is that double mutants of two AGP-specific galactosyltransferase genes (*GALT2* and *GALT5*) and two leucine-rich repeat receptor-like kinase (RLK) genes (*FEI1* and *FEI2*) phenocopy this mutant of *AtSOS5*/*AtFLA4*, respectively [110,116]. It has been demonstrated that these five proteins act linearly in the same signaling pathway of cellulose synthesis, in which AtSOS5/AtFLA4, glycosylated by GALT2 and GALT5 in the Golgi, helps to sense turgor pressure and transmits signals to plasma membrane-localized FEI1 and FEI2 [116]. An in-depth study on FEI1 and FEI2 brings ACC into play, where inhibition of ACSs suppresses the expansion defect in *fei1 fei2* mutant by the disruption of an ethylene-independent pathway. As FEIs do not alter ACS activity and FEIs interact directly with ACS5 in a nonphosphorylation-dependent manner, it has been proposed that FEIs may form a scaffold to localize ACS or may complex ACS with other proteins and that ACC itself may act as a signaling molecule in cellulose synthesis during cell expansion rather than ethylene [110]. Thus, in this model, GPI-anchored AGPs, such as AtSOS5/AtFLA4, may act as a signal sensor to relay information to FEI proteins; then FEI proteins interact directly with ACSs and, as a consequence, collaborate on cellulose synthesis, possibly via an ACC-mediated signaling pathway (Figure 1).

#### 4.2.2. AGPs as Structural Components Affect Cellulose Deposition through Cross-Linking to Other Cell Wall Components

Cellulose associates with hemicelluloses to form a framework embedded in a matrix of pectins and proteins, allow the cellulose microfibrils to move apart during cell wall loosening, and trap them in place when cell wall growth stops [147,176,177]. Pectins, defined as a heterogeneous group of polysaccharides, are major components of the primary cell wall [176,178]. The complex and dynamic pectin network consists of homogalacturonans (HGs), rhamnogalacturonans type I (RG-I), and RG-II, with a small amount of xylogalacturonans, arabinans, and AG I, which are covalently linked to each other [147]. Hemicelluloses are cross-linking polymers of diverse structures, including xyloglucans, xylans, arabinoxylans, mannans, glucomannans, and *β*-glucans [179].

Cell wall components, including polysaccharides cellulose, hemicelluloses, and pectins, as well as structural proteins (such as AGPs, the protagonists of this review), interact covalently and noncovalently to form the functional cell wall [147,148,149,180]. Hijazi et al. proposed an overview of the interactions assumed or demonstrated between HRGPs and cell wall polysaccharides, highlighting the linkages of AGPs with pectins and hemicelluloses and their contribution to cell wall architecture [180]. The classical AGP, AtAGP57C, has been revealed to covalently attach to hemicellulosic and pectic polysaccharides, with RG-I and HG linked to Rha residues in AG polysaccharides and with arabinoxylan attached to either a Rha residue in the RG-I domain or directly to an arabinosyl residue in the AG glycan domain, to form ARABINOXYLAN PECTIN ARABINOGALACTAN PROTEIN1 (APAP1) in *Arabidopsis* cell suspension cultures [96]. AtAGP31 is a nonclassical AGP member with an N-terminus histidine (His)-rich stretch, a repetitive Pro-rich domain, and a C-terminus Cys-rich PAC (PRP and AGP containing Cys) domain [101]. AtAGP31 has been demonstrated to interact in vitro with galactans, which are lateral chains of RG-I through its PAC domain, bind to methylated polygalacturonic acids through its His-rich stretch, and show in vitro self-assembly, providing evidence for the model of noncovalent networks between AGPs and other cell wall components [103].

Arabidopsis seed coat mucilage is an excellent model to study cellulose synthesis and its interactions with other cell wall polymers [165]. AtSOS5/AtFLA4 and FEI2 are found to not only participate in root growth, but also act in a similar pathway to regulate seed coat mucilage synthesis and deposition of cellulose rays during the hydration process of Arabidopsis seeds [110,111]. Previously, AtSOS5/AtFLA4 was suggested to affect cellulose synthesis on the seed coat surface, which, in turn, influences the anchoring of pectin components in seed coat mucilage [111]. However, further studies on *atsos5*/*atfla4* revealed that the formation of cellulosic rays in the adherent mucilage layer was disrupted, with a significantly reduced pectin content, while the cellulose content in mucilage was hardly affected [112,113,165]. The pectin matrix is implicated in the deposition of cellulose microfibrils [181,182]. A hypothesis was proposed that AtSOS5/AtFLA4 could act as a structural component independently of cellulose biosynthesis and signaling, instead organizing cellulose microfibrils through interconnections with pectins or hemicelluloses, and that FEI2 would be required to localize AtSOS5/AtFLA4 in the plasma membrane [112,113,165].

**Figure 2:**
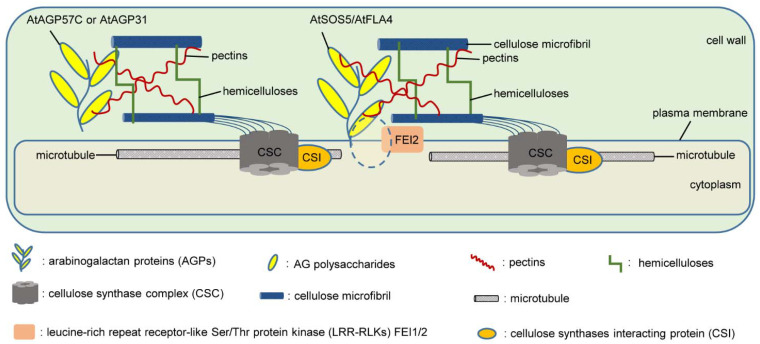
A hypothetical model of AGPs as structural components affecting cellulose deposition through interconnections with other cell wall components, such as hemicelluloses and pectins. AtAGP57C covalently attaches to hemicellulosic and pectic polysaccharides, as proposed by Tan et al. for the APAP1 complex [96]. Noncovalent networks between AtAGP31 and cell wall polysaccharides refer to Hijazi et al. [103]. AtSOS5/AtFLA4 and pectin interconnections in a FEI2-dependent manner are represented according to [112,113,165].

Taken together, we propose a model in which AGPs act as structural components affecting cellulose deposition through interconnections with other cell wall components, such as hemicelluloses and pectins (Figure 2).

#### 4.2.3. AGPs Participate in the Deposition of Cellulose Microfibrils through the Microtubule as an Intermediary

The length, deposition angle, and crystallinity of cellulose microfibrils show a decisive effect on the physical properties of the cell wall [183]. AGPs have been shown to affect cellulose deposition in plant cell walls. In poplar, *PtFLAs* are found to be expressed in the xylem, of which 10 genes are specifically expressed in tension wood (TW). Some of these genes are upregulated in TW (*PtFLA1-10*), which might be related to mechanical properties of TW [184]. Two FLA-encoding genes in *Eucalyptus grandis* W. Hill ex Maiden, *EgrFLA1* and *EgrFLA2*, exhibit higher expression levels in the xylem of TW in the upper sides of branches that possesses a higher cellulose content and a low microfibril angle but, instead, a lower expression level in xylem below these branches, deeply implying an accordance between *FLA* expression level and cellulose content as well as microfibril angle [185]. Arabidopsis *AtFLA11* and *AtFLA12* are highly expressed in stems, mainly distributed in vascular bundles, surrounding parenchyma and vessels. In *Atfla11/fal12* double mutants, the decreased cellulose content leads to a reduction of tensile strength, while the increased cellulose microfibril angle gives rise to a decrease in tensile stiffness, indicating that AtFLA11 and AtFLA12 could interfere with the deposition of cellulose microfibrils during the formation of the secondary cell wall [99].

Cortical microtubules can guide CSCs to move along the microtubule array in a cellulose synthase interactive 1 (CSI1)-dependent manner and, as a consequence, to affect the cellulose microfibril angle [154,155,158,173]. The close linkage between AGPs and cytoskeletal structures, including microfilaments and microtubules, has shed light on the potential role of AGPs in cellulose deposition through a cytoskeletal network. *REB1*/*RHD1* encodes a UDP-D-Glc 4-epimerase, which is involved in the galactosylation of AGPs and xyloglucans [186]. The trichoblasts of mutant *reb1-1* are highly swollen with cortical microtubules that are disordered or even completely absent and lack certain AGP epitopes, suggesting a connection between the organization of cortical microtubules and the deposition of AGPs [186,187]. Sardar et al. demonstrated that *β*-Yariv treatment in tobacco tissue culture cells triggers depolymerization/disorganization of microtubules and F-actin, and cytoskeletal disruptors alter LeAGP1 localization along the Hechtian strands (a stretched plasma membrane extending from the plasmolyzed protoplast to the cell wall in plants), implying that GPI-anchored AGPs play a role in the plasma membrane–cytoskeleton connection [138]. Further evidence that cortical microtubules’ disorganization is induced by *β*-Yariv reagent and two mABs (JIM13 and JIM14) in root epidermal cells substantiates the hypothesis that cell surface AGPs influence the organization of cortical microtubules inside the cell [188]. In addition, the distance between cortical microtubules and the plasma membrane is increased significantly with *β*-Yariv reagent treatment [188]. All these findings lead to the hypothesis that altered AGP status impacts the mechanical properties of the cell wall, transmits the flow of communication from the cell wall to the microtubules by unknown transmembrane protein(s), and results in altered microtubule organization or dissociation from the membrane [138,188].

**Figure 3:**
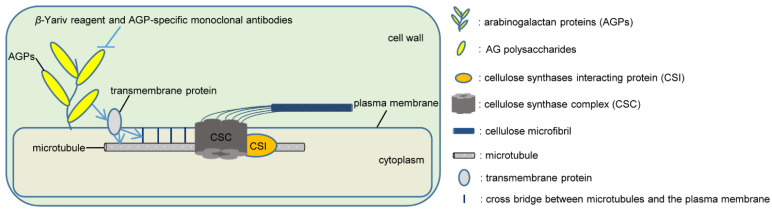
A hypothetical model of AGPs regulating the deposition of cellulose microfibrils by affecting the arrangement of cortical microtubules and/or the connection between cortical microtubules and the plasma membrane through transmembrane protein(s). This model is proposed based on previous studies by Nguema-Ona et al. and Sardar et al. [138,188].

Based on the abnormalities of cellulose deposition in AGP mutants described above, it is speculated that AGPs may regulate the deposition of cellulose microfibrils by affecting the arrangement of cortical microtubules and/or the connection between cortical microtubules and the plasma membrane through transmembrane protein(s) (Figure 3).

#### 4.2.4. AGPs Act as Potential Signal Molecules during Cell Wall Biogenesis

Almost two decades ago, Showalter envisioned some likely scenarios for AGPs in molecular interactions and cellular signaling at the cell surface [2]. Since AGPs are proteoglycans and their protein backbone is decorated by AG polysaccharides, AG polysaccharides determine the characters of AGPs and affect their functions [3,38,39,40], as previously mentioned in this review. So far, a series of evidence has been provided to emphasize the importance of AG polysaccharides for AGP signaling. GhGalT1 is implicated in the biosynthesis of the *β*-1,3-galactan backbone of AGPs and is responsible for the glycosylation of AGPs in cotton [170]. The length of cotton fibers in *GhGalT1* RNAi silencing lines becomes longer. Interestingly, the level of JIM8 (a mAB)-responsive carbohydrates epitopes is decreased [170]. Prolyl 4-hydroxylases in tomato (SlP4Hs) are involved in Pro hydroxylation of AGPs. The level of JIM8-bound epitopes in *SlP4H*-silenced tomato plants is also altered, inferring phenotypes of root tip and branch lengthening and leaf enlargement [189]. This similarity leads to an assumption that particular carbohydrate epitopes related to JIM8 in AGPs may be associated with cell elongation and expansion. In addition, the Arabidopsis mutant *mur1*, with blocked biosynthesis of _L_-fucose in the AG polysaccharides of AGPs, displays a dwarf phenotype and a decreased root cell elongation, implying that AGPs modified by _L_-fucose participate in cell elongation and growth [190]. Defects in the synthesis of AG glycans of AGPs, caused by the functional disruption of KNS/UPEX1 (a type II GALT), results in pollen aggregation and reduced fertility [191]. Furthermore, GlcA residues have also been demonstrated to be essential for the biosynthesis of type II AG and normal function of AGPs [192,193]. The Arabidopsis *β*-glucuronosyltransferases participate in the process of grafting GlcA on AGP glycans. Mutation in *AtGlcAT14A* leads to a reduction of GlcA substitution and an enhanced cell elongation during seedling growth [193]. A knockout mutant of the Arabidopsis *β*-glucuronidase (GUS) gene *AtGUS2*, *atgus2-1*, has decreased GlcA content and shortened hypocotyl, consistent with a role for the AG polysaccharides of AGPs in cell growth [192].

The carbohydrate components of AGPs contain a lot of structural information, which makes potential candidates for chemical signals. The carbohydrate moieties can be extracellularly processed by glycosidases, such as *β*-galactosidases, and detached from AGPs to form free AG glycans, therefore providing the possibility for AGPs in signaling [51,194]. Some excellent reviews have given detailed information of a number of glycoside hydrolases (GHs) involved in the metabolism of AGP carbohydrate moieties, including *β*-galactosidases, *β*-galactanases, *α*-arabinofuranosidases, *β*-arabinopyranosidases, *β*-glucuronidases, *α*-fucosidases, and *α*-rhamnosidases [55,195,196]. However, only a few plant GHs have been reported to hydrolyze AGP glycans relative to the well-characterized AGP-degrading GHs from microbial origin [196], and more exploration is still warranted to understand the role of AG polysaccharide structure towards the AGP function in plant growth and development.

**Figure 4:**
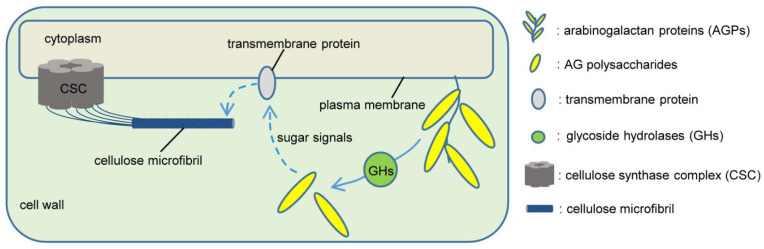
A putative mechanism is an enzymatic release of AG polysaccharides from AGPs that may act as signal molecules possibly recognized by plasma membrane receptors. The sugars may be cleaved by glycoside hydrolases and may function as signal molecules binding to specific receptors, as proposed by Showalter [2].

Plant cells mainly undergo anisotropic growth, including diffusion and tip growth [197]. Cell growth is achieved through strictly controlled cell wall expansion. In this unique process, the influx of water from the extracellular space forms turgor pressure to act on cell wall elasticity and extensibility. Thus, wall stress relaxation may result from the loosening and shifting of load-bearing linkages between cellulose microfibrils. Subsequently, the cell wall expands, and newly synthesized cellulose microfibrils, as well as the pre-existing wall polymers, deposit on the thinned cell wall to further re-form cross-linking with matrix polysaccharides secreted into the wall [147]. The above-mentioned deficient mutants of AG polysaccharides or GlcA residues of AGPs display cell expansion alterations, a phenotype with a delayed elongation and growth. All this is reminiscent of cell expansion, but the deposition process of new cell wall components could be disturbed, resulting in abnormal anisotropic growth of cells. It has been speculated that AGPs may regulate the cellulose deposition process in the cell wall through their AG polysaccharides as signal molecules possibly recognized by plasma membrane receptors [29], thus achieving an anisotropic growth of cells (Figure 4).

#### 4.2.5. AGPs Act as Putative Ca^2+^ Capacitors to Regulate Cellulose Deposition Possibly through Pectin–Ca^2+^ Cross-Links

The carbohydrate moieties of AGPs may not only act as potential chemical signals but also participate in the signal transduction process by chelation with calcium ions (Ca^2+^). AGP6 and AGP11 are two classical AGPs with specific expression and functional redundancy in pollens and pollen tubes [65,66,67,68]. The double null mutant *agp6 agp11* shows phenotypes that include collapsed pollen grains, inhibited pollen tube growth, and precocious pollen germination inside the anthers [67,68]. Costa et al. found that the expression of calcium- and signaling-related genes was altered in *agp6 agp11* pollen tubes, indicating the putative involvement of AGPs in Ca^2+^ signaling cascades [69]. Additional studies have provided evidence for this potential function of AGPs. The AG polysaccharides of AGPs have been verified to bind Ca^2+^ at GlcA residues with a binding stoichiometry of 2:1 at pH = 5, to form an AGP–Ca^2+^ oscillator, thereby activating H^+^ ATPase on the plasma membrane and allowing the influx of Ca^2+^ into cells [198]. AG isolated from *glcat14* triple mutants deficient in the *β*-glucuronosyltransferases that transfer GlcA to the AG has lower Ca^2+^ binding capacity in vitro, and the plants with this defective AG have multiple developmental defects, such as reduced trichome branching, and limited seedling growth [199]. Taken together, these findings imply that the binding of GlcA on AGP polysaccharides to Ca^2+^ is important for cell elongation and growth.

Ca^2+^ signaling is involved in abiotic stress, wound response, stomatal movements, self-incompatibility, interaction with pathogenic microorganisms, tip growth (pollen tube growth and root hair growth), and other vital processes in plants [43,200,201], in which AGPs are also widely involved. This opens the possibility that an AGP–Ca^2+^ oscillator may participate in multiple signal transduction processes in cells. Boron deficiency in *A. thaliana* causes Ca^2+^ influx in root cells and induces the expression of calcium signaling-related genes [202]. It is speculated that boron could interact with Gal residues in the GPI anchor structure of AGPs to stabilize the anchorage of AGPs to the plasma membrane. At the same time, GPI anchors could be used as boron receptors to release Ca^2+^ by an AGP–Ca^2+^ oscillator in the periplasm after sensing boron deficiency and then initiate a series of downstream signal transduction processes [203]. Like AGPs, auxin is also implicated in many processes of plant growth and development, and it is also capable of triggering an intracellular Ca^2+^ signal response [204]. Based on these findings, Lamport et al. proposed a novel concept of an AGP–Ca^2+^–auxin signaling cascade model: first, auxin-activated plasma membrane H^+^-ATPase could release H^+^, thus lowering extracellular pH; subsequently, AGP–Ca^2+^ oscillator would release Ca^2+^ that enters the cytosol through Ca^2+^ channels; then, Ca^2+^ recycled from the cytosol via Golgi vesicle exocytosis would recharge the AGP capacitors to form a reservoir again [43].

In addition to acting as a Ca^2+^ reservoir, AGPs may also associate with RLKs to mediate various signaling transductions [92]. The Arabidopsis AtENDOL14 is a GPI-anchored AGP with a plastocyanin-like domain, which has strong and specific physical interaction with the extracellular domain of FERONIA [94]. As a plasma-membrane-localized receptor kinase, FERONIA has been recently proved to induce Ca^2+^ signaling to maintain cell wall integrity during salt stress [205]. The trio of AtENDOL14, FERONIA, and Ca^2+^ signaling suggests a possibility that GPI-anchored AGPs are involved in FERONIA-dependent Ca^2+^ signaling [92,205].

Ca^2+^ is found in the cell wall ionically cross-linked to HGs in the pectin matrix [147]. In the presence of Ca^2+^, pectin cross-linking Ca^2+^ occurs to form the “eggbox” structure, which has been proposed to be load-bearing components in cell walls [206]. It has been demonstrated that pectins can bind to cellulose during its synthesis and deposition through interactions with, for example, Ca^2+^-deficient regions of HGs and binding of the arabinan and galactan side chains to the cellulose, and these bindings are reversible [207,208]. After Ca^2+^ chelation, pectin cross-linking Ca^2+^ may be removed and pectins recycled [69,209]. In addition, a recent study indicates that the strength of the pectin–Ca^2+^ hydrogels affects cellulose structure, crystallinity, and material properties [209].

**Figure 5:**
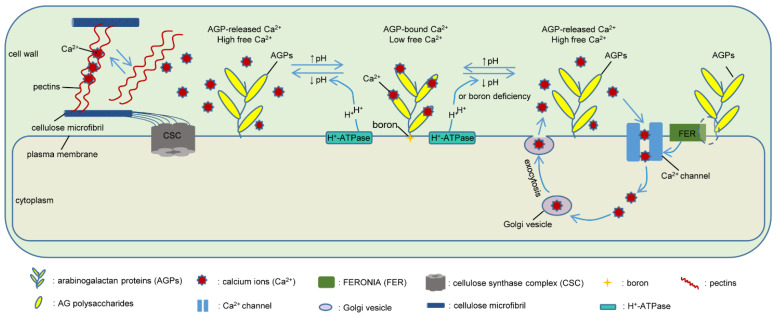
A hypothetical model of AGPs acting as putative Ca^2+^ capacitors to regulate cellulose deposition in the cell wall possibly through pectin–Ca^2+^ cross-links. The AGP–Ca^2+^ oscillator refers to Lamport et al. [43].

Since AGPs have a higher affinity for Ca^2+^ than pectin, a discharged AGP–Ca^2+^ capacitor would be recharged by Ca^2+^ recycled from the cytosol and possibly from the wall matrix (e.g., Ca^2+^–pectin) [198]. Cellulose/Ca^2+^-bound pectin interactions and the novel concept of dynamic Ca^2+^ recycling by an AGP–Ca^2+^ oscillator underlie an interesting possibility that AGPs may act as putative Ca^2+^ capacitors to regulate cellulose deposition possibly through pectin–Ca^2+^ cross-links (Figure 5).

## 5. Conclusions

A number of features including the functional redundancy of AGP family members, the complex post-translational modification process involving many related genes, a high complexity of the carbohydrate side chain structure, and the inability of *β*-Yariv reagent to recognize a single specific AGP hinder our complete understanding of this gene family. We have been continuously looking for links in numerous research studies on AGPs and trying to find clues that can reasonably explain the functional mechanisms of AGPs in vital activities in plants, as well as connecting these data to compile a possible mechanistic scenario. On the basis of previous studies, five models of how AGPs may participate in cellulose synthesis and deposition during cell wall biogenesis have been proposed: (A) AGPs sense extracellular signals by carbohydrate side chains and transmit signals to some receptor kinases, thereby regulating cell wall formation by promoting cellulose synthesis through an ethylene-independent ACC pathway; (B) AGPs serves as structural components affecting cellulose deposition through cross-linking to other cell wall components, such as hemicelluloses and pectins; (C) AGPs regulate the deposition of cellulose microfibrils by affecting the arrangement of cortical microtubules and/or the connection between cortical microtubules and the plasma membrane through transmembrane protein(s); (D) AGPs act as potential chemical signals with their AG polysaccharides; and (E) AGP–Ca^2+^ oscillator forms by chelating Ca^2+^ to regulate cellulose deposition in the cell wall possibly through pectin–Ca^2+^ cross-links. These hypothetical models can provide some clues for further research on the functions of AGPs in cellulose synthesis and deposition, without discarding other mechanistic pathways that might also be involved. Since members from different AGP subfamilies have fairly distinct characteristic domains, the exact molecular mechanisms of AGP action in complicated plant biological processes, not solely devoted to cellulose metabolism and deposition, will certainly require further in-depth investigations in the near future.

## Figures and Tables

**Table 1:** List of AGPs implicated in diverse plant growth and development processes.

Gene ^a^	Species	Classification	GPI Anchor ^b^	Subcellular Localization	Expression Pattern	Genetic Analysis	Phenotype	Biological Function	References
*AtAGP4*/*JAGGER*	*Arabidopsis thaliana* (L.) Heynh.	classical AGP	√	–	stigma, style, transmitting tract, and ovules	T-DNA insertion mutant and RNA interference (RNAi)	polytubey block and persistent synergid	blocks pollen tube attraction	[63,64]
						overexpression	aborted ovules and seeds		
*AtAGP6* and *AtAGP11*	*A. thaliana*	classical AGP	√	–	pollen and pollen tubes	T-DNA insertion single mutant	no discernible phenotype	have overlapping functions in pollen and pollen tube development	[65,66,67,68,69]
						T-DNA insertion double mutant and RNAi	collapsed pollen, inhibited pollen tube growth, and untimely pollen germination		
*BcMF8*	*Brassica campestris* L.	classical AGP	√	plasma membrane, extracellular spaces, and cell walls	pollen and pollen tubes	antisense RNA	sunken pollen with abnormal intine, decreased pollen germination, and retarded pollen tube growth	contributes to pollen wall development, aperture formation, and pollen tube growth	[70,71]
*BcMF18*	*B. campestris*	classical AGP	√	plasma membrane, extracellular spaces, and cell walls	pollen	antisense RNA	shrunken and withered pollen with abnormal cellulose distribution, lacking intine, cytoplasm, and nuclei	required for microspore development and pollen intine formation	[72,73]
						ectopic overexpression	reduced male fertility, short siliques with low seed set, aborted pollen grains without all cytoplasmic materials and nuclei, and no cellulose accumulation in intine		
*AtAGP40*	*A. thaliana*	AG peptide	√	–	pollen	T-DNA insertion mutant	no alteration in pollen grain development but a reduction in pollen grain fitness	prevents premature pollen grain germination	[74]
						*agp6 agp11 agp40* triple mutant	a significant reduction in seed production and a higher number of early germinating pollen tubes inside the anthers		
*Gsp-1*	*Triticum aestivum* L.	AG peptide	–	probably inside vacuoles	developing endosperms	RNAi	increased grain hardness and decreased viscosity of aqueous extracts	required for endosperm formation	[75]
*TTS*	*Nicotiana tabacum* L. and *N. alata* Link & Otto	non-classical AGP	×	extracellular matrix	stylar transmitting tissue	antisense RNA and sense cosuppression	reduced pollen tube growth and reduced female fertility	functions in growth and guidance into the ovules of the pollen tubes	[76,77,78]
*Na120K*/*NaPRP5*	*N. alata*	nonclassical AGP	×	extracellular matrix	styles	RNAi	unable to perform *S*-specific pollen rejection but retains the ability to reject *N. plumbaginifolia* pollen	functions in *S*-specific pollen rejection (self-incompatibility)	[79,80,81,82]
*AGPNa3*/*RT35*	*N. alata*	nonclassical AGP	×	–	stigma	–	–	has a specific, yet to be determined, role in the pistil	[83]
*AtFLA3*	*A. thaliana*	FLA	√	plasma membrane	pollen and pollen tubes	RNAi	shrunken and wrinkled pollen grains with abnormal cellulose distribution in intine	involved in microspore development and may affect pollen intine formation	[84]
						overexpression	defective elongation of the stamen filament, reduced female fertility, wrinkled rosette leaves, more rapid primary root growth, and abnormal root cap cells		
*AtFLA5* and ***AtFLA10***	*A. thaliana*	FLA	√	–	ovules	–	–	may be related to embryogenesis and seed development	[85,86,87]
*AtFLA14*	*A. thaliana*	FLA	√	plasma membrane and Hechtian strands	pollen	T-DNA insertion mutant	no discernible phenotype but precocious pollen germination inside the mature anthers under high moisture conditions	required for pollen development and preventing premature pollen germination under high humidity	[88]
						overexpression	abnormal pollen grains with a shrunken and withered appearance, reduced fertility, short mature siliques, and lower seed set		
***AtFLA9***	*A. thaliana*	FLA	√	–	seedlings, flowers, and siliques	T-DNA insertion mutant	enhanced seed abortion under control conditions; impaired embryo development	plays a role in embryo development, seed setting and response to drought stress	[89,90]
						gain-of-function	reduced seed abortion under drought conditions and increased abortion under control conditions; impaired embryo development		
*BrFLA2*, *BrFLA28*, and *BrFLA32*	*B. rapa* L.	FLA	√	plasma membrane and Hechtian strands	anthers, pollen, and pollen tubes	RNAi	precocious pollen germination in the anthers under high humidity	indispensable for the proper timing of pollen germination under high relative humidity	[54]
*AtENODL9*	*A. thaliana*	*ENODL*	√	plasma membrane	vascular system in leaves, stems, and roots	T-DNA insertion mutant	a significant reduction in the overall reproductive potential	involved in the reproduction process	[91,92]
*AtENODL11*, *AtENODL12*, ***AtENODL13*, *AtENODL14*/*AtEN14***, and *AtENODL15*/*AtEN15*	*A. thaliana*	*ENODL*	√	*AtENODL14* and *AtENODL15*: plasma membrane and filiform apparatus	*AtENODL11* and *AtENODL12*: flowers, fruits, and embryo sacs; *AtENODL13*, *AtENODL14*, and *AtENODL15*: seedlings, roots, flowers, ovules, and stomatal lineage cells	T-DNA insertion single and double mutants	no obvious phenotypes	*AtENODL11*–*AtENODL15*: functionally redundant in pollen tube reception; *AtENODL13*, *AtENODL14*, and *AtENODL15*: required for stomatal lineage development	[33,85,86,89,93,94,95]
						*enodl13-1*; *enodl14-1*; *enodl15-1* triple mutant	significant defects in stomatal patterning and defects in division regulation		
						*en13 en14 en15* triple mutant	no obvious phenotypes		
						*en*-*RNAi* mutant	remarkably reduced seed set, aborted ovules, and failure of pollen tube burst		
						*EN15* overexpression	disturbed pollen tube guidance and reduced fertility		
*AtAGP57C*/*APAP1*	*A. thaliana*	classical AGP	√	cell walls	–	T-DNA insertion mutant	higher inflorescence stem and reduced covalent linkages in cell walls	involved in maintaining wall architecture	[96]
*AtFLA11/IRX13* and *AtFLA12*	*A. thaliana*	FLA	√	–	inflorescence stems	T-DNA double mutant	altered cell wall architecture with increased cellulose microfibril angle and reduced cellulose content and altered stem tensile strength and stiffness	contributes to secondary cell wall formation	[97,98,99]
*AtFLA16*	*A. thaliana*	FLA	×	plasma membrane and cell wall	hypocotyls of young seedlings, roots, rosette leaves, stems, flowers, and siliques	T-DNA insertion mutant	reduced stem length, reduced first internode length, fewer rosette leaves, altered carbohydrate content and biomechanics	involved in stem elongation and secondary cell wall synthesis and function	[100]
*AtAGP31*	*A. thaliana*	nonclassical AGP	×	–	vascular bundles	–	–	may be involved in vascular tissue function during defense response and development	[101,102,103]
***AtXYP1*** and ***AtXYP2***	*A. thaliana*	XYLP	√	–	*AtXYP1*: cotyledons, roots, anthers, and pistils; *AtXYP2*: vasculature, roots, inflorescences, and stems	T-DNA insertion double mutant	defects in vascular development: discontinuous veins, improperly interconnected vessel elements, and simplified venation	involved in vascular development	[52,53,86]
*AtAGP14*	*A. thaliana*	AG peptide	√	plasma membrane	endodermis, root hair zone	T-DNA insertion mutant	markedly increased length of root hairs under control and phosphate (Pi)-deficient conditions	regulates root hair elongation exhibiting environmental response behavior	[104]
*AtAGP15* and *AtAGP21*	*A. thaliana*	AG peptide	√	plasma membrane–apoplastic space	–	T-DNA insertion mutant	*apg21*: aberrant root hair development; *apg15*: a milder phenotype than *apg21*	involved in root development	[105]
*AtAGP30*	*A. thaliana*	nonclassical AGP	×	–	roots	T-DNA insertion mutant	inhibited root regeneration in vitro and suppression of the ABA-induced delay in germination	plays a role in root regeneration, seed germination, and ABA response	[106,107]
						overexpression	severely affected shoot development		
***AtFLA1***	*A. thaliana*	FLA	√	–	stomata, trichomes, anthers, embryos, and roots	T-DNA insertion mutant	increased lateral roots and reduced shoot regeneration in an in vitro shoot induction assay	plays a role in lateral root development and shoot regeneration	[85,86,89,108]
***AtSOS5*/*AtFLA4***	*A. thaliana*	FLA	√	plasma membrane, Hechtian strands, and apoplast	roots, leaves, stems, flowers, siliques, and seed coat	T-DNA insertion mutant and ethyl methane sulfonate-induced mutant	abnormal cell expansion, thinner walls, reduced middle lamella in response to salt stress, and reduction in cellulose across the seed mucilage inner layer	maintains root growth under salt stress and involved in the formation of seed mucilage	[38,86,109,110,111,112,113,114,115,116]
*AtFLA18*	*A. thaliana*	FLA	×	–	all organs, including leaves, stems, siliques, and flowers	T-DNA insertion mutant	short and swollen lateral roots and slightly longer primary root when grown on sensitizing condition of high-sucrose containing medium	plays a role during root elongation	[117]
						*fla18*-*sos5* double mutant	a more severe perturbation of anisotropic growth in both lateral roots and primary roots, a small, chlorotic shoot phenotype under restrictive conditions		
*BcFLA1*	*B. carinata* A. Braun (Ethiopian mustard)	FLA	√	plasma membrane and cell wall	roots	CRISPR/Cas9	reduced root hair length in inorganic Pi-deficient conditions	has a predicted role in the Pi deficiency-induced root hair elongation	[60]
*CsAGP1*	*Cucumis sativus* L.	classical AGP	√	–	most vegetative tissues	overexpression	taller stature and earlier flowering	involved in stem elongation	[118]
*PtaAGP6*	*Pinus taeda* L.	Lys-rich AGP	√	cell walls or extracellular spaces	wood, shoot tips, pollen cones, roots, and planings	–	–	functions in xylem differentiation and wood formation	[119]
*PtFLA6*	*Populus trichocarpa* Torrey & A. Gray ex Hooker	FLA	–	–	xylem tissues of stems	antisense RNA	inhibited tension wood formation in the upper side and enhanced GA3 biosynthesis and GA signaling	plays important roles in GA-mediated tension wood formation	[120]
*ZeXYP1*	*Zinnia elegans* L.	XYLP	√	–	meristem, procambium, and xylem	–	–	mediates local and inductive cell–cell interactions required for xylem differentiation	[52,53,121,122]
*GhFLA1*	*Gossypium hirsutum* L.	FLA	√	cell walls	fibers	RNAi	reduced fiber initiation and elongation, leading to shorter mature fibers	involved in fiber initiation and elongation	[123,124]
						overexpression	promoted fiber elongation		
*GhAGP4*	*G. hirsutum*	FLA	√	–	fibers	RNAi	inhibited fiber initiation and elongation, shorter fiber length, worse fiber quality, and affected cytoskeleton network and cellulose deposition of fiber cells	essential for the initiation and elongation of cotton fiber development	[125,126]
*AtAGP18*	*A. thaliana*	Lys-rich AGP	√	plasma membrane and Hechtian strands	roots, stems, flowers, and leaves	RNAi	functional megaspore fails to enlarge and mitotically divide	functions in plant growth and development, female gametogenesis, and determining megaspore fate	[50,127,128,129,130,131]
						overexpression	smaller rosettes, shorter stems and roots, more branches, less viable seeds, and abnormal maintenance of surviving megaspores		
*AtAGP19*	*A. thaliana*	Lys-rich AGP	√	–	roots, flowers, stems, seedlings, leaves, and siliques	T-DNA insertion mutant	smaller, rounder, and flatter rosette leaves, lighter-green leaves containing less chlorophyll, delayed growth, shorter hypocotyls and inflorescence stems, fewer siliques, and less seed production	functions in various aspects of plant growth and development, including cell division and expansion, leaf development, and reproduction	[50,130,132,133]
*LeAGP-1*	*Lycopersicon esculentum* Mill.	Lys-rich AGP	√	plasma membrane and Hechtian strands	roots and stems	overexpression	multiple branches and less seeds	functions in plant growth and development, probably by linking the plasma membrane to the cytoskeleton	[134,135,136,137,138]
						transgenic tobacco BY-2 cells treated with *β*-Yariv	terminal cell bulging, puncta formation, disturbed microtubule organization, and actin filament formation		
*attAGP*	*L. esculentum*	classical AGP	√	plasma membrane and cell wall	precisely at the site of dodder attack	RNAi and virus-induced gene silencing	reduced attachment force of *Cuscuta reflexa* to host tomatoes	promotes the parasite’s adherence	[139]
*AtAGP17/RAT1*	*A. thaliana*	Lys-rich AGP	√	plasma membrane and Hechtian strands	roots, stems, flowers, and leaves	T-DNA insertion mutant	resistant to *Agrobacterium tumefaciens* root transformation	allows Agrobacterium rapidly to reduce the systemic acquired resistance response during infection	[129,140,141]
						overexpression	no phenotype		
*NaAGP4*	*N. alata*	Lys-rich AGP	√	–	roots, stems, flowers, and leaves	–	–	responds to wounding and fungal infection	[142]
*AtAGP24*	*A. thaliana*	AG peptide	√	plasma membrane	pollen, roots, and siliques	overexpression	enhanced disease susceptibility to the fungus	involved in the pathogen response; may be involved in regulating cell separation in floral abscission zones	[35,85,143,144]
***AtFLA8/AtAGP8***	*A. thaliana*	FLA	√	–	roots, leaves, flowers, and ovules	T-DNA insertion mutant	significantly increased susceptibility to root-knot nematode *Meloidogyne incognita*	plays a role in defense against root-knot nematodes	[85,86,87,89,145]
*GhAGP31*	*G. hirsutum*	nonclassical AGP	×	cell walls	roots, hypocotyls, and ovules	overexpression	improved freezing tolerance of yeast cells and cold tolerance of Arabidopsis seedlings	responses to cold stress during early root development	[146]

^a^ Confirmed GPI-anchored AGPs from proteomics analysis are in bold [85,86,89]; ^b^ existence of predicted GPI anchors (√, exists; ×, does not exist); dashes represent no data.
